# Isolation and identification of protease-producing *Bacillus amyloliquefaciens* LX-6 and its application in the solid fermentation of soybean meal

**DOI:** 10.3389/fbioe.2023.1226988

**Published:** 2023-07-13

**Authors:** Xinyi Huang, Huijie Li, Tao Han, Jiteng Wang, Zheng Ma, Xiaoping Yu

**Affiliations:** ^1^ Zhejiang Provincial Key Laboratory of Biometrology and Inspection and Quarantine, College of Life Sciences, China Jiliang University, Hangzhou, Zhejiang, China; ^2^ Department of Aquaculture, Zhejiang Ocean University, Zhoushan, Zhejiang, China

**Keywords:** *Bacillus amyloliquefaciens*, protease, solid fermentation, soybean meal, glycinin

## Abstract

Soybean meal (SM) is considered an ideal substitute for fish meal; however, its application is mainly limited because of its antigen proteins, glycinin and β-conglycinin. To improve the value of SM in the aquaculture industry, we employed an aerobic bacterial strain (LX-6) with protease activity of 1,390.6 ± 12.5 U/mL. This strain was isolated from soil samples and identified as *Bacillus amyloliquefaciens* based on morphological and physiological biochemical characteristics and 16S rDNA gene sequence analyses. Subsequently, we quantified the extent of glycinin and β-conglycinin degradation and the total protein and water-soluble protein content after SM fermentation with *B. amyloliquefaciens* LX-6. At 24 h of fermentation, the macromolecular antigen proteins of SM were almost completely degraded; the maximum degradation rates of glycinin and β-conglycinin reached 77.9% and 57.1%, respectively. Accordingly, not only did the concentration of water-soluble proteins increase from 5.74% to 44.45% after 48 h of fermentation but so did the concentrations of total protein and amino acids compared to those of unfermented SM. Field emission scanning electron microscopy revealed that the LX-6 strain gradually disrupted the surface structure of SM during the fermentation process. In addition, *B. amyloliquefaciens* LX-6 exhibited broad-spectrum antagonistic activity and a wide pH tolerance, suggesting its application in SM fermentation for fish meal replacement.

## 1 Introduction

Soybean meal (SM) is a by-product of soybean oil extraction, a process involving drying, dehulling, flaking, expanding, and extraction (Fischer et al., 2001; Van Eenennaam and Young, 2014). SM not only contains high protein content and excellent amino acid (AA) quality but also large amounts of antioxidant compounds such as flavonoids and phenols (Lee et al., 2022). SM is often used as a cheap and high-quality protein raw material for poultry feeding and in livestock industries (Qi et al., 2023). The recent development of the aquaculture industry has resulted in a lack of protein sources for aquaculture feed and more expensive conventional raw materials, such as fish meal (Caimi et al., 2021). To reduce aquaculture costs, SM could serve as an ideal substitute for fish meal. However, its application is limited by its relatively high level of antinutritional factors (ANF) (Karr-Lilienthal et al., 2005), especially the antigen proteins glycinin and β-conglycinin. Known food allergens interfere with digestion, absorption, and nutrient use (Peng et al., 2018), causing hypersensitivity in animals (Hotz and Gibson, 2007; Cai et al., 2015). The presence of ANF seriously affects the use value and application scope of SM.

Microbial fermentation is an effective biotechnological approach to improving the nutritional value of SM by degrading macromolecular proteins and ANF compounds (Frias et al., 2008; Sun et al., 2020). Numerous microorganisms can be used in soybean and soy-based product fermentation and for improving the nutritional quality of SM, including *Rhizopus oligosporus* (Zhang et al., 2022), *Aspergillus oryzae* (Handa et al., 2019), *Lactobacillus* (Sun et al., 2022), and yeast (Luján-Rhenals et al., 2015). Owing to its short growth cycle and safety, *Bacillus* is considered an ideal strain in the fermentation industry. Moreover, SM fermentation with *Bacillus* produces an end product of high nutritional value and low ANF, which has fostered research in this area (Yao et al., 2021). For example, Zheng et al. (2017) fermented SM with *Bacillus siamese*, which degraded the allergic protein and loosened its structure, improving its solubility and animal absorption. Moreover, Jones et al. (2010) investigated the effects of fermentation using several strains, discovering that *B. subtilis* could best degrade antigen proteins and increase the crude protein content. Antigen protein degradation depends on protease hydrolysis; therefore, high protease activity is key for microbial SM fermentation (Yao et al., 2021).

In this study, we screened and identified *Bacillus amyloliquefaciens* LX-6, a strain with high protease activity, which we used to ferment SM. Later, we quantified the degradation of glycinin and β-conglycinin as well as the water-soluble protein and AA contents in SM after fermentation.

## 2 Materials and methods

### 2.1 Source of SM and strains

SM was obtained from Zhejiang Ocean University. Soil samples were collected from different ecological areas in China, such as wetland, forest, waterfall, and lakeshore. Soil samples were dried and stored at 4°C.

### 2.2 Media and cultural conditions

The medium for isolating protease-producing strains comprised (L^−1^ distilled water) 50-g skim milk powder and 20-g agar powder. The medium for determining protease activity comprised the following (L^-1^ distilled water): 40-g SM, 1.5-g CaCl_2_, 4.1-g glucose, 3-g NaCl, and 9.4-g yeast extract. The solid-state fermentation medium comprised the following (L^−1^ distilled water): 30-g SM and 30-mL distilled water in a 250-mL conical flask. The SM was sieved through 20 meshes and added to a solid-state fermentation medium.

### 2.3 Isolation and screening of protease-producing strain

Five grams of soil samples were weighed and added to a 250-mL conical flask containing 45-mL sterile water. Then, dozens of glass beads (4 mm) were added, incubated at 28°C for 2 h at 180 rpm, and immersed in an 80°C water bath for 20 min. Target strains were isolated by serial dilution (diluted gradient to 10^–1^, 10^–2^, and 10^–3^), spread on a skimmed milk plate, and cultured at 37°C for 24 h. Various individual colonies forming hydrolysis zones were streaked onto a skimmed milk plate for further verification and purification. The diameter of the hydrolysis zone in the skimmed milk plate was measured as an indication of the protease activity of the isolated strains. All strains were tested in two independent experiments run in triplicate.

### 2.4 Determination of enzyme activity

Strains with larger hydrolysis circles were cultured in protease fermentation medium with 4% inoculum at 37°C for 48 h at 180 rpm. The protease activity was determined using the Folin-phenol method of the People’s Republic of China (GB/T 23527–2009). The reaction mixture contained 1.0 mL of 2.0% casein, and 1.0 mL of the supernatant was incubated at 40°C for 20 min. After that, the reaction was terminated by adding 2.0 mL of 0.4 mol/mL trichloroacetic acid to the mixture, followed by incubation at 40°C for 20 min. Then, 1 mL of the supernatant of this mixture was mixed with 5 mL of 0.4 mol/mL Na_2_CO_3_ and 1 mL of Folin’s reagent. Finally, the mixture was incubated for another 20 min at 40°C. Tyrosine content was quantified at 660 nm. One unit (U) was defined as the amount of enzyme required to release l-μg tyrosine from the casein per minute under assay conditions.

### 2.5 LX-6 identification

#### 2.5.1 Morphological observation of the LX-6 strain

The protease-producing strain LX-6 was streaked on Luria-Bertani (LB) solid medium and cultured at 37°C for 24 h to observe colony growth. Spores were stained with malachite green using a spore staining kit (Beijing Sorabio Technology Co., Ltd.).

An appropriate 0.05 g strain LX-6 bacterial solution was fixed with 2.5% glutaraldehyde overnight at 4°C. The strain LX-6 suspension was centrifuged at 11,000 × g for 5 min to obtain the cells, which were subsequently washed three times for 15 min with 1 × phosphate buffered saline buffer. Then, cells were fixed in 1% osmic acid for 1 h and gradually dehydrated with 30%, 50%, 75%, 90%, and 100% ethanol for 15 min (Din et al., 2013). Next, samples were dried with a Hitachi HCP-2 critical point dryer and plated in Hitachi B-1010 ion plating units. Strain LX-6 morphology was observed using a Hitachi SU-8010 scanning electron microscope at 6,000× magnification.

#### 2.5.2 Physiological and biochemical characteristics of strain LX-6

The physiological and biochemical characteristics of strain LX-6 were analyzed by 7% sodium chloride growth, Voges-Proskauer (V-P) determination, nitrate reduction, D-mannitol fermentation, and other tests (Pereira et al., 2020; Ma et al., 2022).

#### 2.5.3 Identification of strain LX-6 by 16S rDNA analysis and phylogenetic analysis

The 16S rDNA of the isolated strain LX-6 was amplified using the primers 16S-F (5′-AGA​GTT​TGA​TCA​TGG​CTC​AG-3′) and 16S-R (5′-CTA​CGG​CTA​CCT​TGT​TAC​GA-3′). The PCR mixture comprised 1 μL 16S-F, 1 μL 16S-R, 1 μL template DNA, 12.5 μL GC buffer I (2×), 4 μL dNTPs, 0.25 μL *Ex* taq DNA polymerase, and added sterile water to 25 μL. The amplification procedure was conducted at 94 °C for 5 min, followed by 30 cycles at 94°C for 45 s, 57°C for 30 s, and 72°C for 45 s, with a final elongation step at 72°C for 10 min. The PCR product of the 16S rDNA was purified, sequenced (Tsingke Biotechnology, Hangzhou, China), and deposited in the NCBI database. The sequence was aligned using BLAST analysis, and the molecular phylogenetic tree (neighbor-joining likelihood) was constructed using MEGA 11.

### 2.6 SM fermentation by the isolated strain LX-6

The isolated strain was grown in LB liquid medium at 37°C and 180 rpm. This liquid culture of the isolated strain (10^8^ CFU/mL) was then inoculated into a fermentation medium containing 30-g SM and 30-mL sterile water (1:1 solid-liquid (g/mL) ratio) in a 500-mL Erlenmeyer flask and fermented at 37 C for 60 h. Samples were collected at 12 h intervals until the end of fermentation. Finally, the fermented SM was freeze-dried, ground, screened through a 60-mesh sieve, and used for further analysis.

### 2.7 SDS-PAGE and determination of total protein and water-soluble protein contents

To determine the degradation of macromolecular proteins in the fermented SM at different times, 10% Tris sodium-dodecyl sulfate-polyacrylamide gel electrophoresis (SDS-PAGE) run at 100 V for 120 min was used (Li et al., 2020). The total protein and water-soluble protein content of the samples were determined by the Kjeldahl method (Rizvi et al., 2022) according to GB T6432-2018 and NY/T 1205–2006, respectively. Glycinin and β-conglycinin were analyzed using a commercially available ELISA kit (Hengyuan BioTechnology Co., Ltd., Shanghai, China).

### 2.8 Analysis of AA composition

The AA composition was measured using an automated AA analyzer (Hitachi L8900, Japan).

### 2.9 Structural analysis of SM and fermented SM by field emission scanning electron microscopy

Microstructural observation of fermented SM was performed using a FE-SEM (SU-8010, China) at 3,000×magnification according to the protocol of the Electronic Microscopy Center of China Jiliang University.

### 2.10 Determination of strain LX-6’s antibacterial activity

The antibacterial activity of LX-6 was explored using *Serratia*, *Staphylococcus aureus*, *Candida albicans*, *Escherichia coli*, and *Fusarium oxysporum* f. sp*. cucumerinum* as indicators. Strains were incubated under suitable conditions until the cell/spore concentrations reached around 10^7^ CFU/mL. Indicator plates were prepared by mixing 200 μL of each indicator solution with agar media. The diameter of the inhibition zone was observed after 24–48 h of incubation.

### 2.11 Statistical analysis

All experiments were performed ≥3 times; the results are expressed as the mean ± standard deviation (SD). The students’ t-test was used for statistical analysis.

## 3 Results

### 3.1 Isolation of a strain with high protease activity, LX-6

**FIGURE 1:**
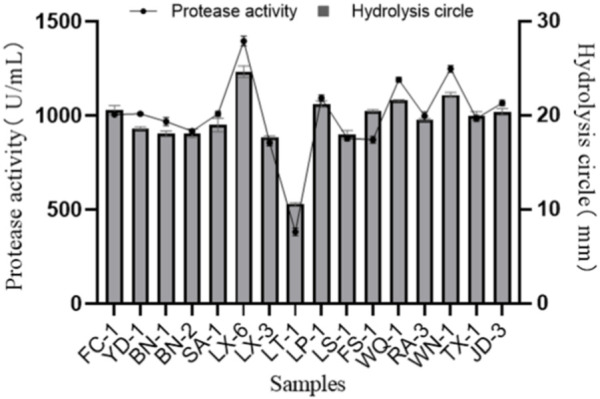
Diameter of the hydrolysis zone and protease activity of 16 representative protease-producing strains. The error bars were calculated from three independent experiments.

Bacterial strains from various samples were directly isolated and screened on a skimmed milk powder plate. A total of 28 isolate-forming hydrolysis zones (diameter > 10 mm) were picked and purified again to verify their protease activity. Among them, 16 strains that exhibited large hydrolysis circles (diameter > 15 mm; Figure 1) were then inoculated into protease fermentation medium for enzymatic activity determination. Among the 16 isolated strains, the strain LX-6 exhibited the highest protease activity, 1,390.6 ± 12.5 U/mL (Figure 1). Because we used different proteins as substrates, the growth status and decomposition efficiency of these strains differed. Therefore, the hydrolytic circle values of 16 isolated strains on skim milk plates were not completely consistent with their protease activity in other media containing different substrates. Finally, strain LX-6 was therefore selected for further experiments.

### 3.2 Strain LX-6 was identified as *Bacillus amyloliquefaciens*

**FIGURE 2:**
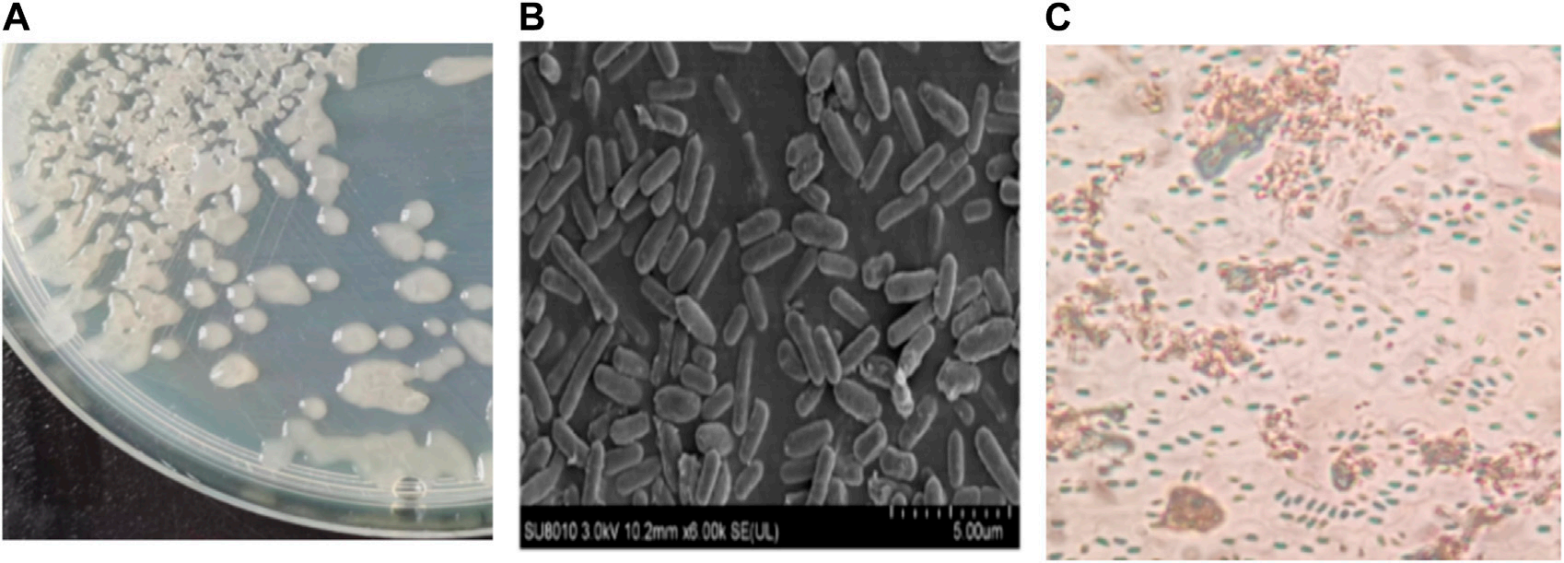
Morphological characteristics of the LX-6 strain. To assess colony morphology, the LX-6 strain was streaked and grown on an LB agar plate, which was incubated at 37°C for 2–3 days **(A)**. Scanning electron microscopy of LX-6 grown in LB medium for 24 h (magnification, ×6,000) **(B)**. Spores were stained with malachite green using a spore staining kit (Beijing Sorabio Technology Co., Ltd.) **(C)**.

**FIGURE 3:**
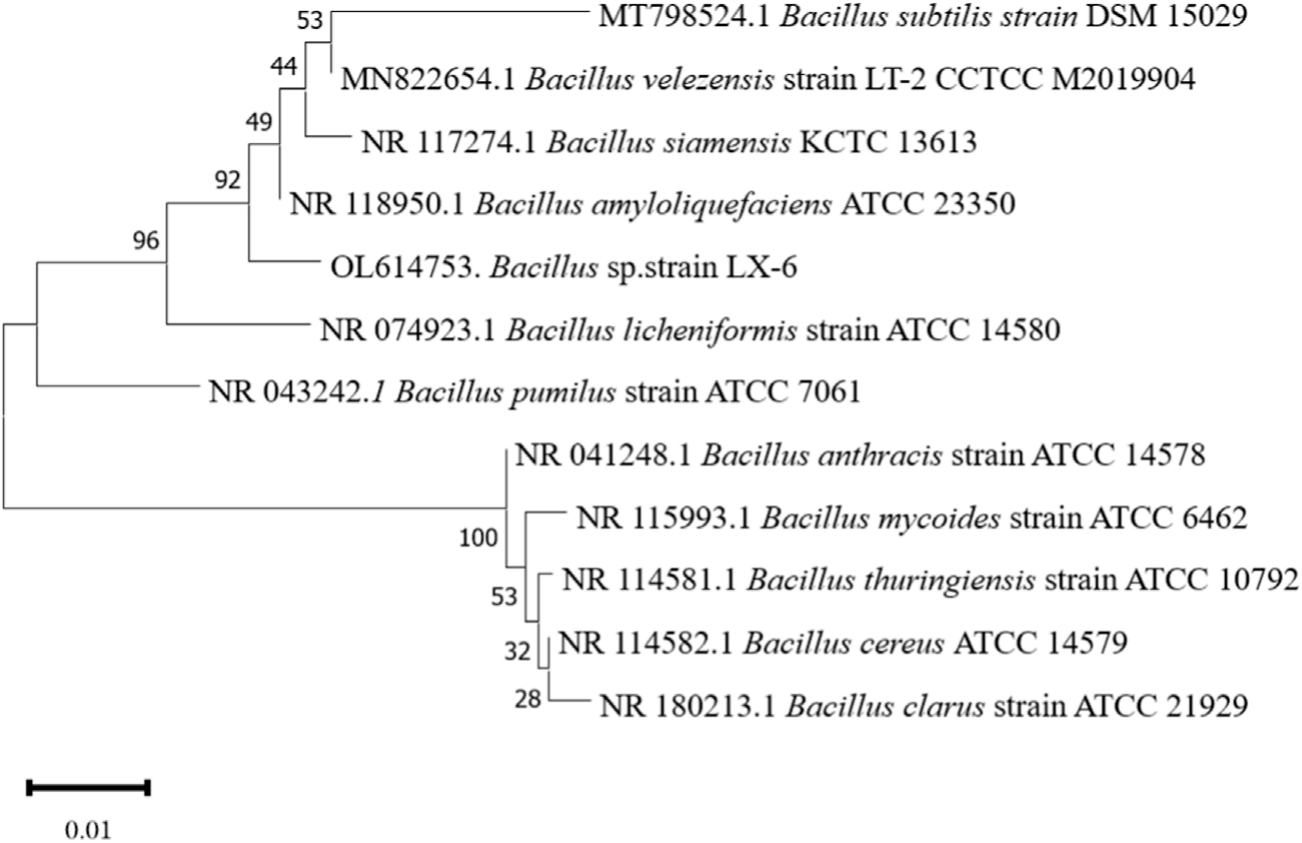
Phylogenetic tree analysis based on the 16S rDNA sequence of the LX-6 strain. Phylogenetic analysis was performed with MEGA 11 using the neighbor-joining method in the Jukes-Cantor model. Bootstrap values (>50%) based on 1,000 replicates are shown at the branch nodes. Bar, 0.01 substitutions per nucleotide position.

The LX-6 colony exhibited a milky white color, a smooth surface, and high viscosity on LB solid plates (Figure 2A). In addition, under SEM, strain LX-6 showed a short rod-shaped morphology (Figure 2B). After staining, spores and cells showed a clear green and red color, respectively (Figure 2C). According to physiological and biochemical assays, strain LX-6 was positive in the V-P, gelatin liquefaction, nitrate reduction, L-arabinose, D-mannitol, and starch hydrolysis tests (Table 1). Therefore, based on its morphological and physiological characteristics, we hypothesized that strain LX-6 belongs to the genus *Bacillus*. Based on 16S rDNA alignment of strain LX-6 and type *Bacillus* strains and their phylogenic evolution analyses, strain LX-6 exhibited the highest similarity (98.34%) with *B. amyloliquefaciens* ATCC 23350 (Figure 3). Accordingly, strain LX-6 was finally identified, named *Bacillus amyloliquefaciens* LX-6, and deposited in the China Center for Type Culture Collection (No. 20211,457). A 1450-bp 16S rDNA fragment of strain LX-6 was cloned, sequenced, and submitted to the GenBank databases under the accession number OL614753.1.

**TABLE 1:** Physiological and biochemical identification of the LX-6 strain.

Physiological and biochemical assays	Results
V-P	−
Citrate	−
Propionate	−
d-xylose	+
l-arabinose	+
d-mannitol	+
Gelatin liquefaction	+
7% sodium chloride growth	−
Nitrate reduction	+
Starch hydrolysis	+
pH range	3–9

### 3.3 SM fermentation with *B. amyloliquefaciens* LX-6 effectively degraded macromolecular antigen proteins

**FIGURE 4:**
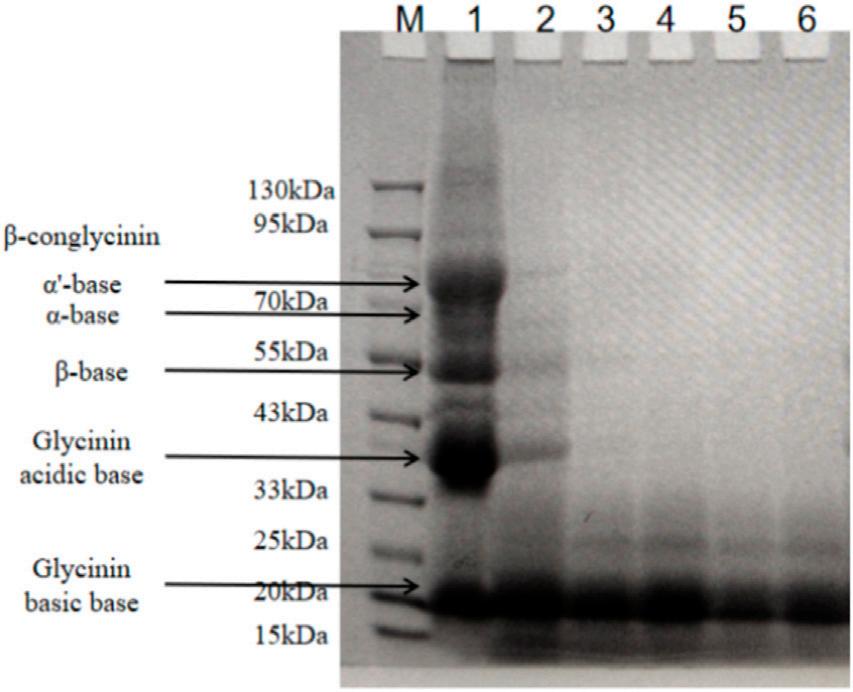
SDS-PAGE analyses of the macromolecular antigen proteins of SM at different fermentation times. Lane M, standard protein molecular weight markers; lane 1, unfermented SM; lanes 2-6, protein profiling of fermented SM with *B. amyloliquefaciens* LX-6 after 12 (lane 2), 24 (lane 3), 36 (lane 4), 48 (lane 5), and 60 h (lane 6) of fermentation at 37°C.

SM contains mainly two types of allergenic proteins: conglycinin (68 kDa α, 72 kDa α’ subunit, and 52 kDa β subunit) and glycinin (37 kDa acidic subunit and 15 kDa basic subunit). We investigated the effects of *B. amyloliquefaciens* LX-6 fermentation on the degradation of glycinin and conglycinin in SM by SDS-PAGE (Figure 4). Some macromolecular antigen proteins started to degrade in the initial stage of fermentation (0–12 h), as indicated by the weaker protein bands (33–90 kDa) of fermented SM than those of unfermented SM. The macromolecular proteins in SM were gradually degraded during fermentation, resulting in successively thinner bands. After 24 h of SM fermentation by *B. amyloliquefaciens* LX-6, macromolecular proteins >20 kDa were almost completely degraded into small molecular products.

### 3.4 Increased total and water-soluble protein contents in fermented SM

**FIGURE 5:**
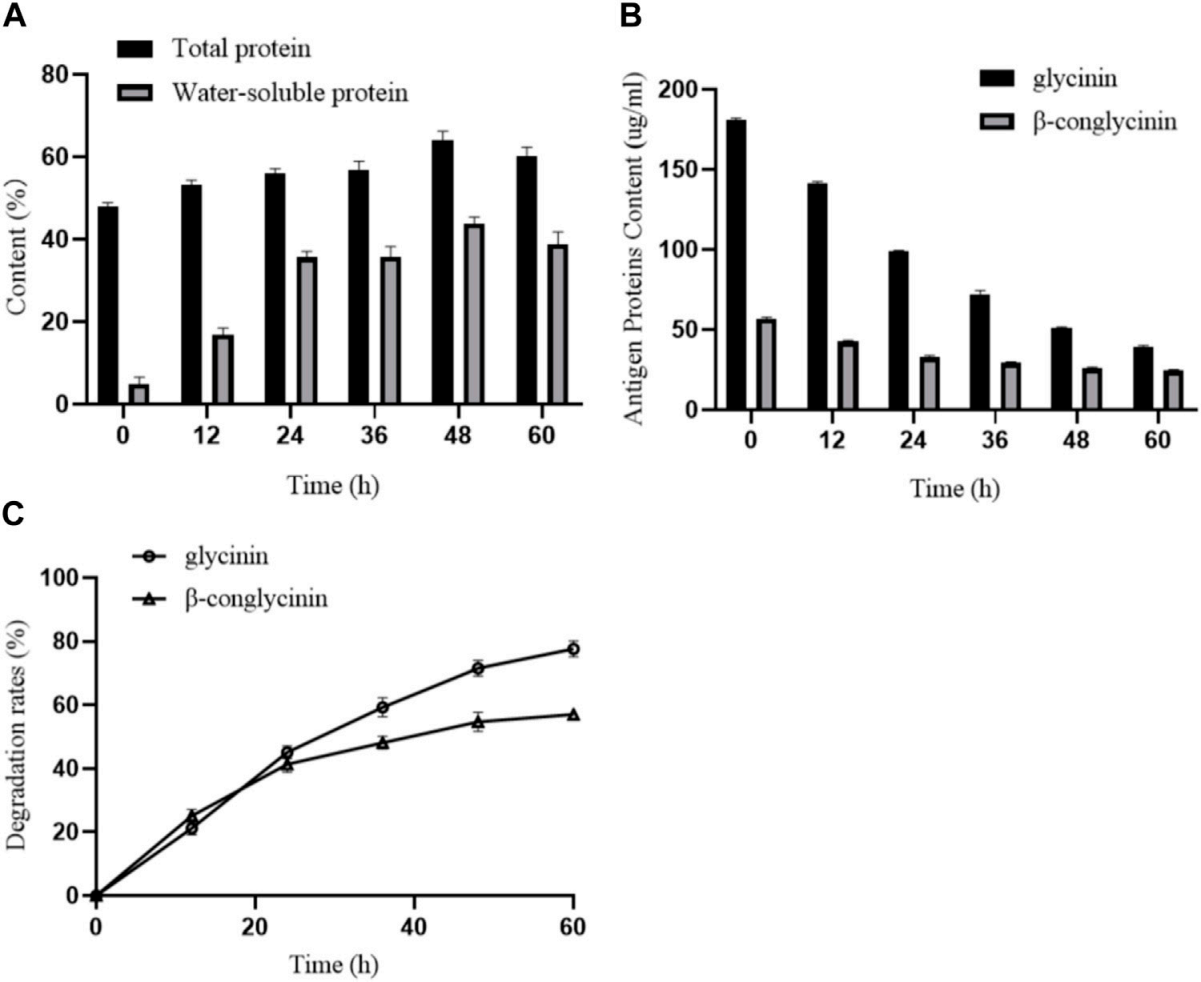
Effect of SM fermentation with *B. amyloliquefaciens* LX-6 on the levels of proteins and degradation of antigenic proteins. SM was inoculated with *B. amyloliquefaciens* LX-6 and fermented at 37°C. The levels of total proteins and water-soluble proteins **(A)**, antigenic protein levels (glycinin and β-glycinin) **(B)**, and degradation rates of glycinin and β-glycinin **(C)** were determined after different periods of fermentation.

To investigate the effects of *B. amyloliquefaciens* LX-6 fermentation on the total protein and water-soluble protein content of SM, we determined the protein contents of unfermented and fermented SM. The total protein content of unfermented SM was 48.87% ± 1.35%, whereas during fermentation by *B. amyloliquefaciens* LX-6, the total protein content gradually increased, reaching a maximum of 64.58% ± 3.07% at 48 h of fermentation (Figure 5A). Similarly, the water-soluble protein content of fermented SM increased significantly, reaching a maximum of 44.45% after 48 h of fermentation, 7-fold higher than that of unfermented SM (Figure 5A). Moreover, after 60 h of SM fermentation by *B. amyloliquefaciens* LX-6, the maximum degradation rates of glycinin and β-conglycinin in SM reached 77.9% and 57.1%, respectively (Figure 5B).

### 3.5 The AA content in SM increased after fermentation

The total AA content increased from 148.4 ± 17.39 mg/mL to 1909.43 ± 387.86 mg/mL after SM fermentation with *B. amyloliquefaciens* LX-6. The levels of essential AA increased more remarkably, from 85.29 ± 8.61 mg/mL to 1,537.72 ± 334.86 mg/mL, than those of nonessential AA. Among essential AAs, the highest increase rate (70-fold) was observed for valine (Val), whose content increased from 15.38 ± 1.26 mg/mL to 1,061.44 ± 220.56 mg/mL, while that of arginine (Arg) decreased after fermentation (Table 2).

**TABLE 2:** Amino acid contents of unfermented SM and SM fermented with *B. amyloliquefaciens* LX-6.

Amino acids		Unfermented SM (mg/mL)	Fermentation with LX-6 for 12 h (mg/mL)	Fermentation with LX-6 for 48 h (mg/mL)
Essential amino acids	Asp	16.98 ± 1.31^b^	10.89 ± 2.54^b^	44.89 ± 10.30^a^
	Thr	6.7 ± 2.40^a^	11.27 ± 3.75^a^	18.82 ± 21.59^a^
	Val	15.38 ± 1.26^b^	237.21 ± 85.91^b^	1,061.44 ± 220.56^a^
	Met	0.39 ± 0.08^c^	6.20 ± 2.39^b^	24.32 ± 1.64^a^
	Ile	1.17 ± 0.07^c^	16.79 ± 6.44^b^	73.20 ± 3.03^a^
	Leu	1.11 ± 0.26^c^	39.26 ± 14.19^b^	133.64 ± 5.02^a^
	Phe	1.42 ± 0.16^a^	31.17 ± 8.83^a^	60.38 ± 42.14^a^
	Lys	4.94 ± 0.12^b^	24.46 ± 7.55^b^	84.65 ± 15.75^a^
	His	2.36 ± 0.23^b^	7.32 ± 3.66^b^	35.63 ± 13.74^a^
	Arg	34.80 ± 2.71^a^	0.36 ± 0.50^b^	0.77 ± 1.08^b^
Total essential amino acids		85.29 ± 8.61^b^	384.91 ± 135.74^b^	1,537.72 ± 334.86^a^
Nonessential amino acids	Glu	45.02 ± 6.48^b^	60.47 ± 24.33^b^	180.76 ± 0.83^a^
	Cys	4.44 ± 0.23^a^	10.14 ± 0.68^a^	21.93 ± 30.31^a^
	Gly	3.45 ± 0.82^b^	3.91 ± 1.25^b^	27.85 ± 2.75^a^
	Ala	8.7 ± 0.89^c^	24.81 ± 4.19^b^	116.99 ± 5.36^a^
	Ser	0.21 ± 0.23^b^	7.08 ± 0.08^a^	5.69 ± 0.92^a^
	Tyr	0	0	0
	Pro	1.25 ± 0.12^a^	0.68 ± 0.43^a^	18.51 ± 12.83^a^
Total amino acids		148.40 ± 17.39^b^	491.98 ± 166.69^b^	1909.43 ± 387.86^a^

### 3.6 Fermentation by *B. amyloliquefaciens* LX-6 altered SM’s microstructure

**FIGURE 6:**
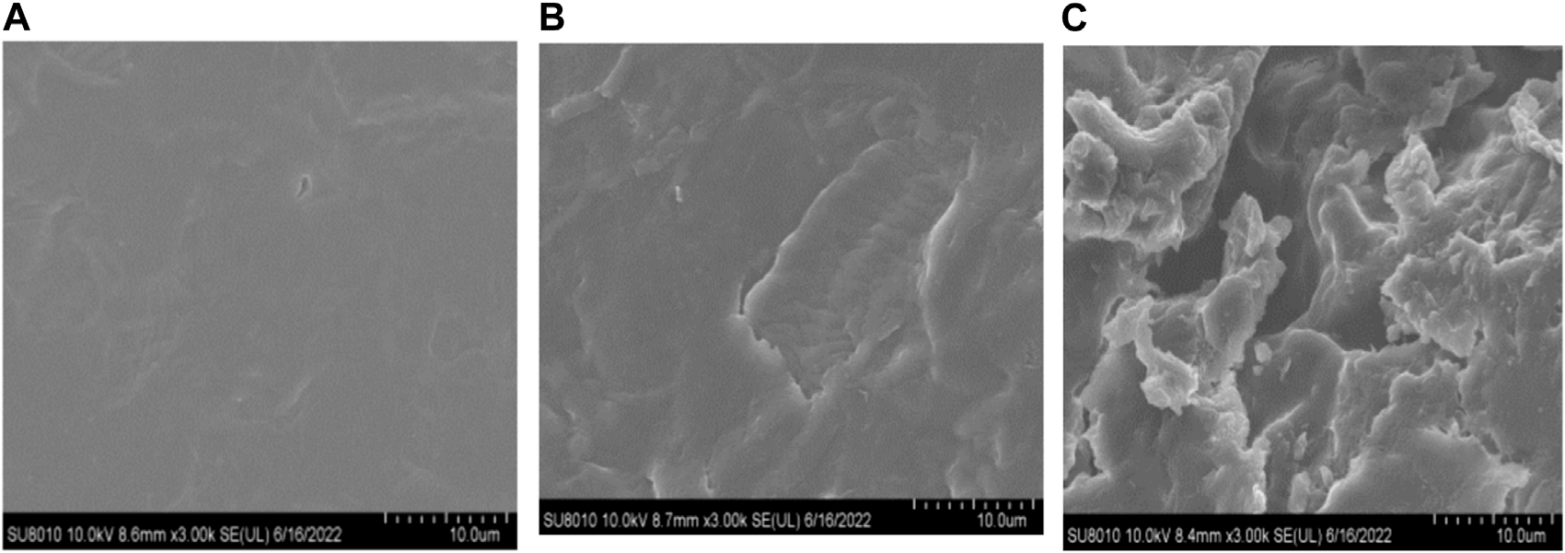
Field emission SEM of unfermented and fermented SM. Unfermented SM **(A)** was inoculated with *B. amyloliquefaciens* LX-6 and fermented at 37°C for 12 **(B)** or 48 h **(C)**. magnification, ×3,000.

FE-SEM was employed to explore the effect of *B. amyloliquefaciens* LX-6 fermentation on SM structure. The structure of the unfermented SM was dense and smooth (Figure 6A). However, after 12 h of fermentation (Figure 6B), the SM microstructure started to show loose and broken elements. After 48 h (Figure 6C), the fermented SM was even more fragmentated and had increased porosity.

## 4 Discussion

**FIGURE 7:**
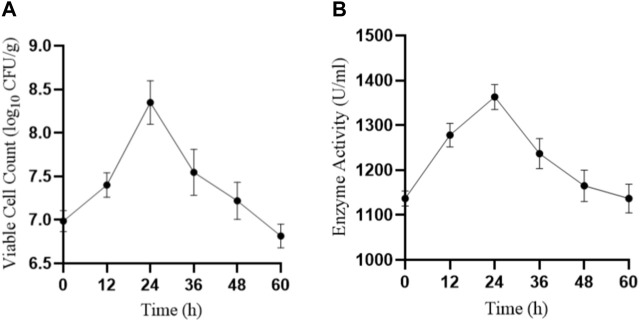
SM was inoculated with B. amyloliquefaciens LX-6 and fermented at 37°C. The viable cell count **(A)** and enzymatic activity **(B)** were determined after different durations of fermentation.

We also tested the viable cell count (Figure 7A) and protease activity (Figure 7B) of LX-6 during different time periods of fermentation SM. Because of *B. amyloliquefaciens* LX-6’s high protease activity, after 24 h of SM fermentation, glycinin and β-conglycinin were effectively degraded as their corresponding bands were almost impossible to detect *via* SDS-PAGE. After 48 h of fermentation, we observed the highest degradation rates of glycinin and β-conglycinin using ELISA. The degradation efficiency of glycinin and β-conglycinin in SM fermented by strain LX-6 obtained in the present study is better than that of previous studies using native *Bacillus* strains (Shikha and Darmwal, 2007; Baweja et al., 2016; Huang et al., 2022), in which the whole fermentation process for full glycinin and β-conglycinin degradation required ≥60 h. The increase in low-molecular protein levels indicated decreased potential allergenicity of SM proteins.

The degradation of SM antigenic proteins increased total protein, water-soluble protein, and AA contents. In comparison, the soluble protein in SM increased only by 10.14% after fermentation with *B. siamensis* (Zheng et al., 2017) and by 8.42% and 7.43% when using *B. sanfenicol* SQVG18 and SQVG22 (Gao et al., 2020). It has been reported that essential AAs are the main limiting factors for SBM replacement of fish meal in aquaculture (Lin and Mui, 2017; Gao et al., 2020). After fermentation with LX-6, the total essential AA levels of SM were 17-fold higher than those of unfermented SM; this increased bio-availability has great potential for replacing fish meal. Excessive SM fermentation causes not only a pungent smell but can also decrease the nutritional value of SM (Medeiros et al., 2018). Accordingly, after 48 h of SM fermentation with *B. amyloliquefaciens* LX-6, the total and water-soluble protein contents decreased slightly, possibly because small proteins were degraded to ammonia or NH_3_ due to excessive protease hydrolysis during the fermentation process. Therefore, future research should focus on the optimization of fermentation conditions for improving SM fermentation efficiency with strain *B. amyloliquefaciens* LX-6. Moreover, SEM revealed that *B. amyloliquefaciens* LX-6 effectively destroyed the SM microstructure, degrading internal macromolecules and increasing nutritional value and digestibility.

Previous phylogenetic analyses revealed that *Bacillus velezensis*, *Bacillus amyloliquefaciens*, and *Bacillus siamensis* clustered tightly to form the “operational group *Bacillus amyloliquefaciens*” (Fan et al., 2017), widely used in the food industry and SM fermentation because of their safety and ability to produce proteases. For example, *B. amyloliquefaciens* ZB has 694 U/g of protease activity and has thus been used for SM fermentation (Wattanakul et al., 2021). The *B. siamensis* isolate JL8 (protease activity 519·1 U/g) was used in the laboratory production of fermented SM, which showed better properties than unfermented SM (Zheng et al., 2017). *B. velezensis* 157, exhibiting 236.94 U/g of protease activity, was mixed with *Lactiplantibacillus plantarum* to degrade ANFs and improve SM nutrition for animal feed via two-stage solid-state fermentation. The protease activity of LX-6 observed in this study (1,390 ± 12.5 U/mL) is much higher than that of the above native or wild-type *Bacillus* strains. Nevertheless, the protease activity of *B. amyloliquefaciens*/*B. velezensis* (Li et al., 2023) could be increased to 20000–30000 U/mL by strategies such as protease purification (Yu et al., 2019), modification of the native strain (Zhou et al., 2020), overexpression or heterologous expression of the *npr* gene encoding protease (Wang et al., 2016), and optimization of fermentation conditions (Guo et al., 2022). Therefore, SM fermentation with *B. amyloliquefaciens* LX-6, a strain with high protease production, can improve its efficiency through future genetic modification, mixed fermentation, and medium optimization.

**FIGURE 8:**
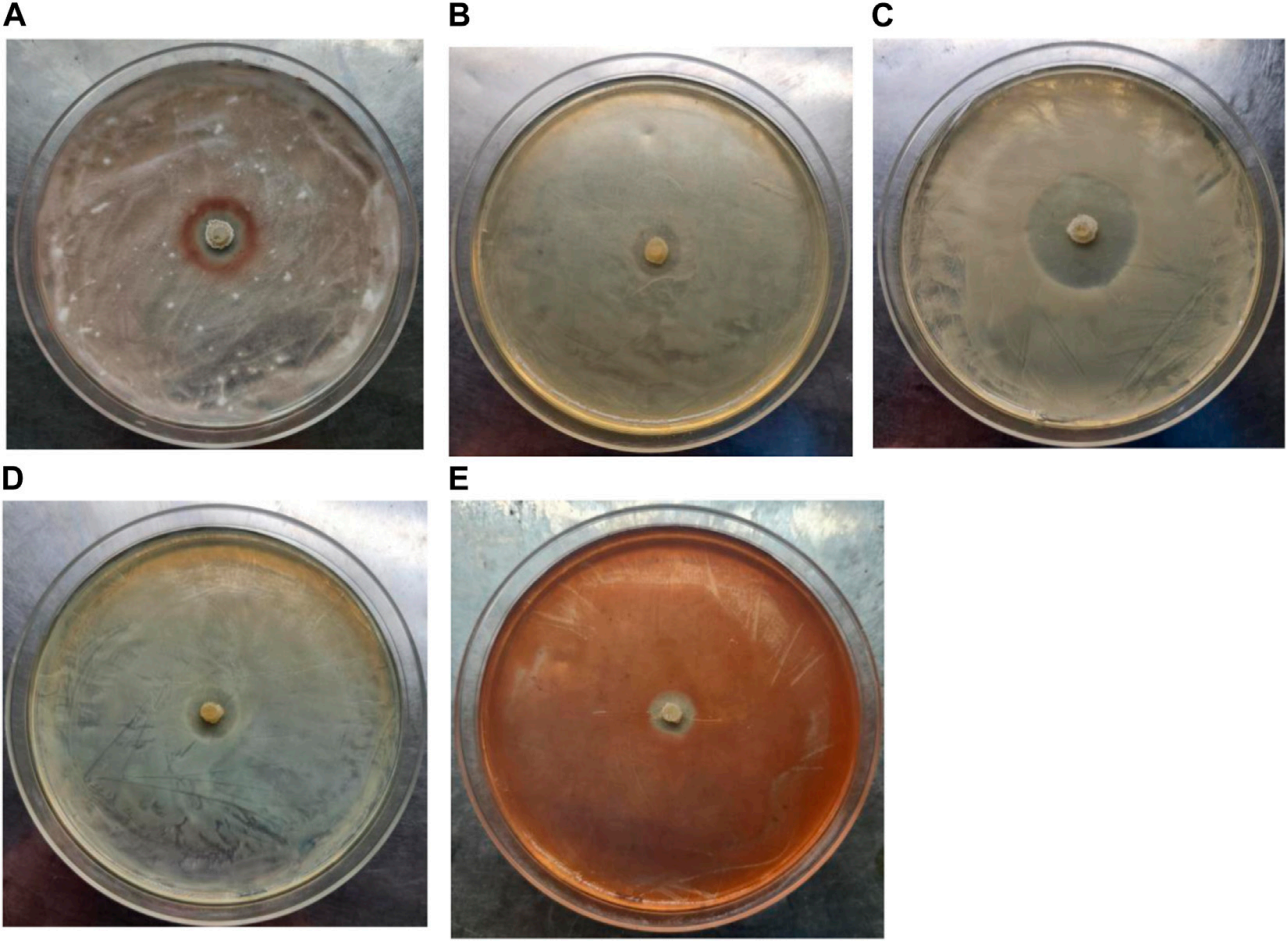
Determination of the antibacterial activity of *B. amyloliquefaciens* LX-6. *F. oxysporum* f. sp. *cucumerinum* **(A)**, *E. coli* **(B)**, *C. albicans* **(C)**, *S. aureus* **(D)**, and *Serratia* **(E)** were used as indicators.

In addition, the antibacterial activity of strain LX-6 was explored using the following five pathogens as indicators: *Fusarium oxysporum* f. sp. *cucumerinum*, *Escherichia coli*, *Candida albicans*, *Serratia*, and *Staphylococcus aureus*. Strain LX-6 had broad-spectrum inhibitory effects on the five tested indicators, exhibiting the strongest inhibition activity against *C. albicans* (inhibition zone reaching 20 ± 1.7 mm; Figure 8), suggesting a certain degree of safety for the use of strain LX-6 in SM fermentation. However, as we did not characterize the presence of the antifungal or antibacterial metabolite substances of LX-6, this aspect warrants further research. In addition, we investigated the feasibility of using *B. amyloliquefaciens* LX-6 under various fermentation conditions and found that it had a broad growth range of pH 3–9 (Table 1), suggesting good suitability for SM fermentation.

In conclusion, an efficient protease producer, *B. amyloliquefaciens* strain LX-6, was isolated and identified. SM fermentation using *B. amyloliquefaciens* strain LX-6 resulted in effective degradation of the antigenic proteins glycinin and β-conglycinin in SM, which increases digestibility while inhibiting pathogenic bacteria and has broad application in aquaculture.

## Data Availability

The datasets presented in this study can be found in online repositories. The names of the repository/repositories and accession number(s) can be found below: https://www.ncbi.nlm.nih.gov/nuccore/OL614753.1.
